# The Rise of the Veterinary Profession

**Published:** 1900-01

**Authors:** T. A. Donaldson

**Affiliations:** Manchester, Conn.


### THE RISE OF THE VETERINARY PROFESSION.

By T. A. Donaldson, V.S.,
MANCHESTER, CONN.

I am not aware of any honorable profession that has had more barriers to its progress than the veterinary profession, especially in the United States.

Before veterinary colleges were instituted the blacksmith shop was the bearer of information for all the ills and treatment of our domestic animals. The farrier (iron worker) became the title of the original horse-doctor. The errors of the iron horse-doctors have been so indelibly grafted into the minds of the people that the horseshoer yet has a grip on a credulous public. The horseshoers still claims the heritage, and, like the silversmiths of old, yet cry out "Great is Diana." This error immigrated to this country. The customs of the average American prove that we are yet to a great extent a conservative people. The blacksmith shop to-day, even in the New England States, to the most conservative is the pool of Siloam to relieve the suffering of our dumb animals. There is not a graduate in the land who does not have this experience, and find some who prefer the traditional and infallible remedies promulgated by our great ancestors, when bleeding was resorted to for every ill, a salt herring run down the throat, split frogs for local applications to the legs; restoring an artificial cud to a cow was a rare virtue. In fact, all skill was a birth-gift from above. The farrier would bleed before he would diagnose. He used the fleam to get fame. He would bleed the horse to bleed the owner. How long will it take to remove this delusion? This conserva-

[1] Offered at the American Veterinary Medical Association, New York City, September, 1899.

tive spirit is not limited to the illiterate, and to prove this I have only to call your attention to what Congress did with army bill. If one State in the Union granted veterinary legislation on the first application, I would like to know what State it is. And the States that have regulated veterinary medicine and surgery are far in the minority. The old State of Connecticut, although appealed to several times, never considered it right to protect the unwary public from imposition nor the graduate who came out on legitimate principles. What a conservative people we are to leave pretenders at liberty so long. How long will it be at this rate before the veterinary surgeons in the United States Army will rank above the position of a common soldier? What a vain boast we hear about the rapid strides of civilization, science, and art. If we are a progressive people, why does our Legislators not encourage us with better protection? And without proper protection the aspiring student has no impetus to go to college and attain proficiency at a large expense. If medical and surgical skill is a good thing for the safety of human life and the protection of live-stock, why, then, is it so repulsive to our wise legislators to give us protection and the encouragement we so justly deserve? Can you mention a country outside of the United States where non-professionals are at liberty to impose on a credulous public as they do here? A century ago veterinary colleges were in the days of their infancy. Since that time it has been a task, that is yet undone, to educate the people to science; and what is the prospect of the future? We first had the iron horse, now we have the trolley, and I understand that the Pope Manufacturing Company has an order for 6000 horseless carriages for this city. The trolley has removed no less than 1,000,000 horses from Uncle Sam's farm. Although these are not favorable to the veterinary profession, they are links in the march of progress and perfectly consistent with the laws of evolution. The department stores will, for they have already, adopted the horseless carriages for the delivery of their goods, and the great interest that is now taken in the improvement of roads will greatly favor their adoption. What inventive genius will not do in the near future is a risky statement to make.

In the face of all these substitutions, threatening the anihilation of the horse, there is enough left to sustain the profession for an untold period. New developments may create new uses for the horse, besides, the services of veterinary surgeons are rapidly increasing in demand for the bovine and ovine herds and flocks, and their services in canine practice is on the increase. The American continent, in my opinion, will be the greatest live-stock nation on the earth.

The wealthy class will never abandon the use of the horse. There is no means of conveyance that affords as much pleasure as to ride in a carriage drawn by a span of proud-looking horses. A ride on the train, on the trolley, on the horseless carriage is monotonous; but the best people in the country never get tired looking at the carriage-horse. The deer is called the forest beauty, but the horse is the beauty of the plain in all historic ages. His proud crest and his flowing mane are attractions of all who are gifted with a keen sense of the beautiful. The admiration of the horse has never depreciated since he was so perfectly described in the glowing language of Job : "His neck is clothed with thunder, he rejoiceth in his strength, and he paweth in the valley." Nothing but selfish economy will ever abandon the use of the horse, and if the American people will continue to use the horse because they are conservative I, for one, will be willing to pardon them.

The student who goes to college with any other purpose in view than to continue a student in the veterinary field, ever searching for knowledge, has sadly missed his calling. The colleges are deserving of much praise for untiring energy in preparing students to treat humanely the order of creation that cannot speak for themselves. And I say with all sincerity that if none were allowed to practise but those who can treat humanely and on scientific principles it would be a greater boon to humanity and live-stock than all the benefits derived from the humane societies. The man who pretends to have skill, if not in the posession of it is an impostor. I am in favor of any kind of liberty that is congenial to our welfare. The cruelties that are inflicted on dumb animals by non-professionals are horrible to relate, and none but the graduate is capable of knowing this ; and to escape the persecution that would follow it is not reported. I would be glad to see the day with a universal law that would allow none to practise but those who can practise on the principles of science.

---

Dr. George M. Waldrod, of Storn Lake, Iowa, writes of an outbreak of glanders in Buena Vista County, Iowa, in which State Veterinarian Gibson and his assistant, Dr. Hammond, already have destroyed some thirty-four horses. The disease seemed to be introduced through the movement of horses incidental to construction of a raiload through that territory.

